# TACE plus donafenib and immune checkpoint inhibitors for intermediate HCC (CHANCE2410 study): a propensity score matching analysis

**DOI:** 10.1186/s41747-026-00748-5

**Published:** 2026-06-16

**Authors:** Rong Ding, Xiao-Yang Xu, Rui-Bao Liu, Jun Tie, Xu-Hua Duan, Bin Xiong, Deng-Gao Yuan, Wei-Jun Fan, Lu Wang, Zhi-Qiang Wu, Jie Zheng, Hui Zhao, Chang-Long Hou, Jin-Long Song, Ben-Sheng Zhao, Xiao-Li Zhu, Yong-Jie Su, Song Wang, Guo-Wen Yin, Li Chen, Hai-Dong Zhu, Gao-Jun Teng, Bin-Yan Zhong, CHANCE2410 Investigators, Rong Ding, Xiao-Yang Xu, Rui-Bao Liu, Jun Tie, Xu-Hua Duan, Bin Xiong, Deng-Gao Yuan, Wei-Jun Fan, Lu Wang, Zhi-Qiang Wu, Jie Zheng, Hui Zhao, Chang-Long Hou, Jin-Long Song, Ben-Sheng Zhao, Xiao-Li Zhu, Yong-Jie Su, Song Wang, Guo-Wen Yin, Li Chen, Hai-Dong Zhu, Gao-Jun Teng, Bin-Yan Zhong, You Lu, Qing-Yu Xu, Hao Jiang, Qing-Qiao Zhang, Wei-Dong Wang, Peng Song, Bo-Gen Ye, Zhong-Wei Zhao, Feng-Zheng Zhang, Zhi-Qiang Fu, Chan Xie, Hai-Min Chen, Qing-He Tang, Ping Guo, Ming-Xin Pan, Jin-Hua Song, Wei-Zhong Zhou, Chun Lv, Shan-Zhi Gu, Kang-Shun Zhu, Ming-Rong Cao, Ning Huang, Hong-Wen Zhang, Qing-Han Li

**Affiliations:** 1 Center of Interventional Radiology and Vascular Surgery, Nurturing Center of Jiangsu Province for State Laboratory of AI Imaging & Interventional Radiology (Southeast University), Department of Radiology, Zhongda Hospital, Medical School, Southeast University, Nanjing, China; 2 National Innovation Platform for Integration of Medical Engineering Education (NMEE) (Southeast University), Nanjing, China; 3 Basic Medicine Research and Innovation Center of Ministry of Education, Zhongda Hospital, Southeast University, Nanjing, China; 4 State Key Laboratory of Digital Medical Engineering, Southeast University, Nanjing, China; 5 Department of Minimally Invasive Intervention, Yunnan Cancer Hospital, The Third Affiliated Hospital of Kunming Medical University, Kunming, China; 6 Department of Vascular Surgery and Interventional Department, The Fourth Affiliated Hospital of Soochow University, Dushu Lake Hospital, Suzhou, China; 7 Department of Interventional Radiology, The First Affiliated Hospital of Soochow University, Suzhou, China; 8 Department of Interventional Radiology, The Tumor Hospital of Harbin Medical University, Harbin, China; 9 National Clinical Research Center for Digestive Diseases and Xijing Hospital of Digestive Diseases, Air Force Medical University, Xi’an, China; 10 Department of Interventional Radiology, The First Affiliated Hospital of Zhengzhou University, Zhengzhou, China; 11 Department of Interventional Radiology, The First Affiliated Hospital, College of Medicine, Zhejiang University, Hangzhou, China; 12 Ningbo No.2 Hospital, University of Chinese Academy of Sciences Ningbo Huamei Hospital, Ningbo, China; 13 Department of Imaging and Intervention, Cancer Center, Sun Yat-sen University, State Key Laboratory of Oncology in Southern China, Guangzhou, China; 14 Fudan University Shanghai Cancer Center, Shanghai, China; 15 Department of Interventional Radiology, The First Affiliated Hospital of Sun Yat-sen University, Guangzhou, China; 16 Department of Interventional Radiology, The First Affiliated Hospital of Wenzhou Medical University, Wenzhou, China; 17 Department of Interventional & Vascular Surgery, Affiliated Hospital of Nantong University, Medical School of Nantong University, Nantong, China; 18 Department of Interventional Radiology, Anhui Provincial Hospital, The First Affiliated Hospital of the University of Science and Technology of China, Hefei, China; 19 Department of Surgical Oncology (Interventional Therapy), Shandong Tumor Hospital and Institute, Jinan, China; 20 Department of Interventional Radiology, The First Affiliated Hospital of Anhui Medical University, Hefei, China; 21 Department of Hepatobiliary Surgery, Zhongshan Hospital of Xiamen University, Fujian Provincial Key Laboratory of Chronic Liver Disease and Hepatocellular Carcinoma, Xiamen, China; 22 Department of Interventional Radiology, The Affiliated Hospital of Qingdao University, Qingdao, China; 23 Department of Interventional Radiology, Jiangsu Cancer Hospital & Jiangsu Institute of Cancer Research & The Affiliated Cancer Hospital of Nanjing Medical University, Nanjing, China; 24 Department of Interventional Radiology, Zhejiang Cancer Hospital, Hangzhou Institute of Medicine (HIM), Chinese Academy of Sciences, Hangzhou, China; 25 Department of Interventional Radiology, The Affiliated Hospital of Xuzhou Medical University, Xuzhou, China; 26 Department of Interventional Radiology, The Affiliated Wuxi People’s Hospital of Nanjing Medical University, Wuxi, China; 27 Department of Interventional Therapy, National Cancer Center/National Clinical Research Center for Cancer/Cancer Hospital and Shenzhen Hospital, Chinese Academy of Medical Sciences and Peking Union Medical College, Shenzhen, China; 28 Renji Hospital, Shanghai JiaoTong University School of Medicine, Shanghai, China; 29 Department of Interventional Radiology, Zhejiang University Lishui Hospital, The Fifth Affiliated Hospital of Wenzhou Medical University, Lishui Central Hospital, Lishui, China; 30 Department of Interventional Radiology, Anhui Provincial Hospital, The First Affiliated Hospital of University of Science and Technology of China (USTC), Hefei, China; 31 Department of Hepatobiliary Surgery, Sun Yat-sen Memorial Hospital, Sun Yat-sen University, Guangzhou, China; 32 The Third Affiliated Hospital of Sun Yat-sen University, Guangzhou, China; 33 Yuebei People’s Hospital, Shaoguan, China; 34 Department of Biliary and Pancreatic Surgery, East Hospital Affiliated to Tongji University in Shanghai, Shanghai, China; 35 The First Affiliated Hospital of Xiamen University, School of Medicine, Xiamen University, Xiamen, China; 36 Zhujiang Hospital of Southern Medical University, Guangzhou, China; 37 Hepatobiliary Center, Jiangsu Provincial People’s Hospital, Nanjing, China; 38 Department of Interventional Radiology, Jiangsu Provincial People’s Hospital, Nanjing, China; 39 Huai’an No.4 People’s Hospital, Huai’an, China; 40 Department of Interventional Therapy, Hunan Cancer Hospital and the Affiliated Cancer Hospital of Xiangya School of Medicine, Central South University, Changsha, China; 41 Department of Interventional Radiology, The Second Affiliated Hospital of Guangzhou Medical University, Guangzhou, China; 42 The First Affiliated Hospital of Jinan University, Guangzhou Overseas Chinese Hospital, Guangzhou, China; 43 Fujian Medical University Union Hospital, Fuzhou, China; 44 The 900 Hospital of the Joint Service Support Force of the People’s Liberation Army of China, Fuzhou, China; 45 Department of Hepatobiliary Surgery, First People’s Hospital of Foshan, Foshan, China

**Keywords:** Carcinoma (hepatocellular), Chemoembolization (therapeutic), Donafenib, Immune checkpoint inhibitors, Prognosis

## Abstract

**Objective:**

To compare the efficacy and safety of transarterial chemoembolization (TACE) plus donafenib and immune checkpoint inhibitors (ICIs) (combination therapy) *versus* TACE monotherapy for intermediate hepatocellular carcinoma (HCC).

**Materials and methods:**

This nationwide, multicenter, retrospective cohort study included intermediate HCC patients receiving either combination therapy or TACE monotherapy between January 2021 and May 2024. The primary outcome was progression-free survival (PFS). The secondary outcomes included overall survival (OS) rate, objective response rate (ORR), and safety. Propensity score matching (PSM) analysis was employed to minimize bias. The Cox proportional-hazards regression model was used to analyze factors affecting PFS and OS.

**Results:**

Of 364 patients enrolled, 192 received combination therapy and 172 received TACE monotherapy with a baseline up-to-seven distribution (≤ 7 = 30%, > 7 = 70%). After PSM, 127 pairs were analyzed. Median PFS was significantly longer for the combination therapy than for TACE monotherapy (19.6 months [95% confidence interval, CI 14.9‒24.4] *versus* 15.3 months [95% CI 12.8–17.8], hazard ratio 0.647 [95% CI 0.464–0.903], *p* = 0.010). The combination therapy group showed better OS rate (94.8% *versus* 83.5%, 1-year OS rate; 76.4% *versus* 64.8%, 2-year OS rate; hazard ratio 0.542 [95% CI 0.327‒0.898], *p* = 0.016) and ORR (78.9% *versus* 62.5%, *p* = 0.002). Grade 3 or 4 adverse events from any cause were 12.5% in the combination group *versus* 5.5% in the monotherapy group. Multivariate analysis identified combination therapy as an independent prognostic factor for both longer PFS and OS.

**Conclusion:**

TACE plus donafenib and ICIs offer superior PFS and OS, supporting its use as an alternative option for intermediate HCC.

**Relevance statement:**

The study highlights the efficacy of the combination therapy of TACE plus donafenib and ICIs in the real world. It has the potential to act as an alternative for the treatment of intermediate HCC with an acceptable safety profile.

**Key Points:**

- Large real-world studies evaluating the combination therapy of TACE plus donafenib and ICIs for intermediate HCC patients are scarce.
- The combination therapy significantly improved PFS, OS, and ORR compared to TACE monotherapy, with a manageable safety profile.
- TACE plus donafenib and ICIs could serve as an alternative option for intermediate HCC.

**Graphical Abstract:**

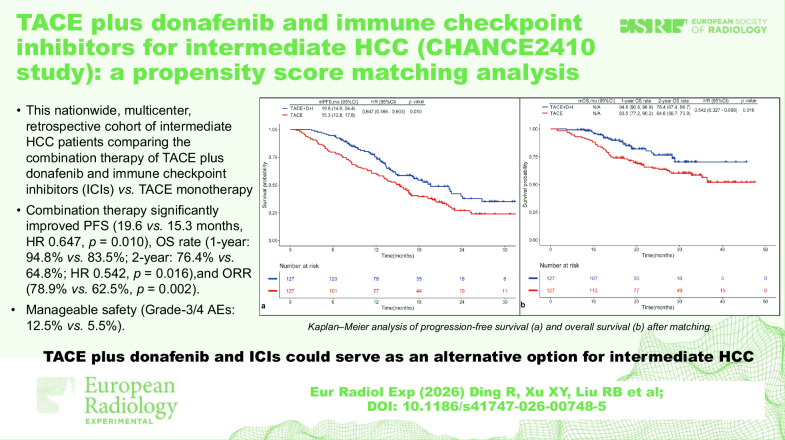

## Background

Transarterial chemoembolization (TACE) remains the standard treatment for patients with intermediate hepatocellular carcinoma (HCC) according to the Barcelona Clinic Liver Cancer (BCLC) staging system [1–3]. TACE works by embolizing the tumor’s blood supply arteries, leading to ischemia and hypoxia in the tumor, thereby inhibiting tumor growth [4, 5]. However, its efficacy is often limited by tumor recurrence and incomplete necrosis. In recent years, combining TACE with systemic therapies, particularly immune checkpoint inhibitors (ICIs) and tyrosine kinase inhibitors (TKIs), has shown promising synergistic effects by enhancing anti-tumor immunity and improving treatment outcomes [6, 7]. Because TACE alters the tumor immune microenvironment, triggering tumor cell necrosis, releasing neoantigens, and activating dendritic cells [8]. These changes can shift the immune microenvironment from an immunosuppressive state, unfavorable for immune checkpoint inhibitors (ICIs), to one that promotes immune activation. Several pivotal trials, such as EMERALD-1 and LEAP012 trials, have shown that TACE combined with ICIs and anti-vascular endothelial growth factor antibodies or TKIs significantly improves progression-free survival (PFS) while maintaining a manageable safety profile compared to TACE monotherapy in intermediate HCC patients [9, 10]. Real-world studies, like the CHANCE series, further support the benefit of TACE combined with ICIs and TKIs over monotherapy in unresectable HCC [11–13].

Donafenib is an oral multi-kinase inhibitor that targets several receptor kinases, including vascular endothelial growth factor receptor, platelet-derived growth factor receptor, and Raf kinase [14, 15]. It has been demonstrated to have superior overall survival (OS) compared to sorafenib and has been recommended as a first-line systemic treatment for advanced HCC in China [16, 17]. TACE plus donafenib also had a short therapeutic onset time, showing a high objective response rate (ORR), as well as controllable toxicity [18, 19].

Currently, large real-world studies evaluating the combination therapy of TACE plus donafenib and ICIs for HCC patients in BCLC stage B are scarce. Therefore, this retrospective study aimed to evaluate the safety and efficacy of TACE plus donafenib and ICIs for patients with intermediate HCC in a real-world setting.

## Methods

### Patient selection

**Fig. 1:**
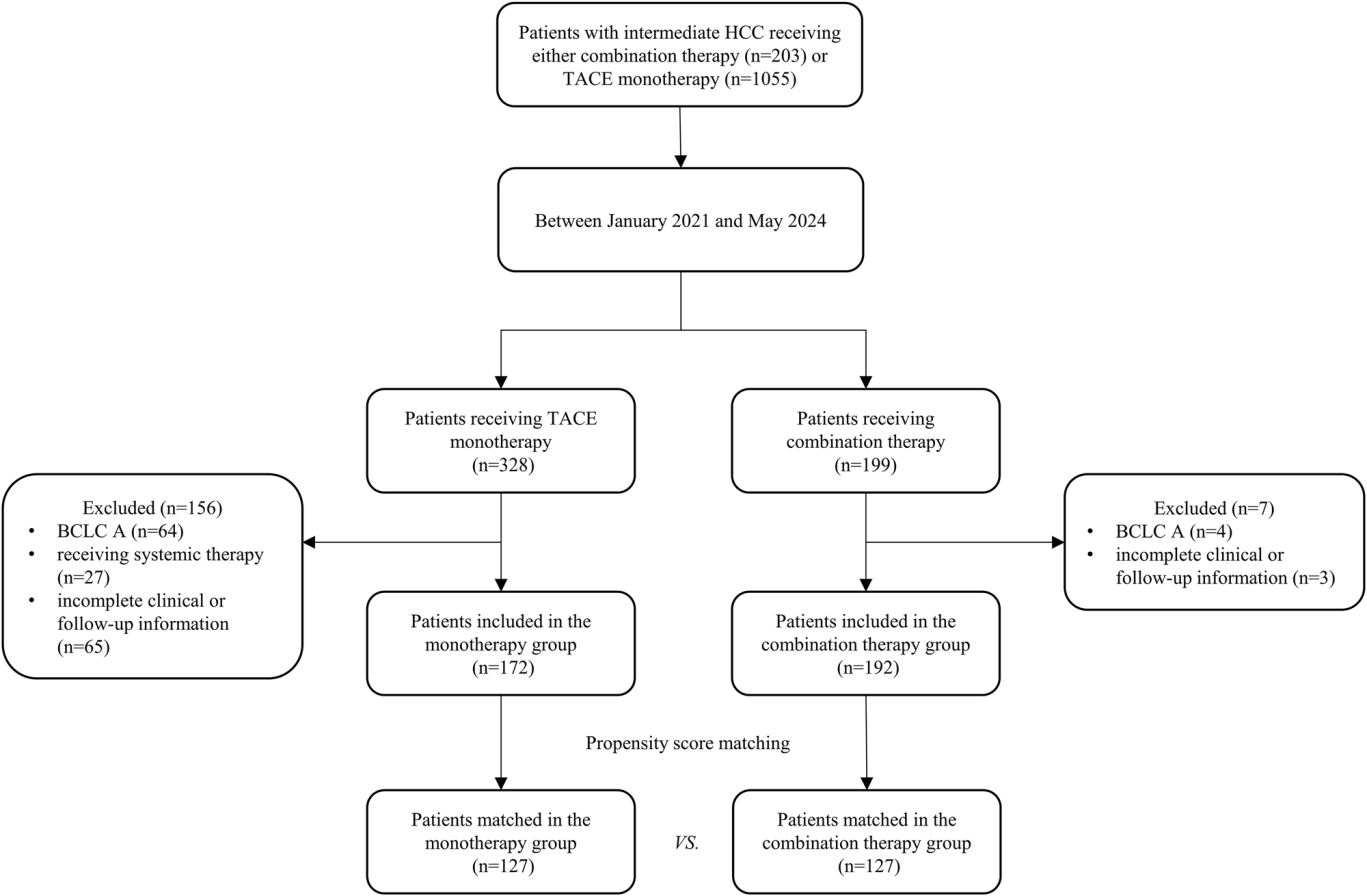
Flowchart of study design

This nationwide, multicenter, retrospective study was approved by the Institutional Review Board and Human Ethics Committees of the participating hospitals. Requirement for informed consent was waived due to the retrospective nature of the study. Patients who received either the combination therapy of TACE plus donafenib and ICIs or TACE monotherapy between January 2021 and May 2024 in 38 participating hospitals of China were screened and enrolled (Fig. 1).

Inclusion criteria were as follows: (1) histologically or clinically confirmed diagnosis of HCC according to the guidelines; (2) BCLC stage B; (3) received either TACE plus donafenib and ICIs or TACE monotherapy; (4) Child-Pugh grade A or B without presence of uncontrollable ascites or hepatic encephalopathy; (5) Eastern Cooperative Oncology Group (ECOG) performance status score 0.

Exclusion criteria were as follows: (1) previously received either TACE or hepatic arterial infusion chemotherapy‒HAIC; (2) previously received systemic therapy such as ICIs, molecular targeted therapies or chemotherapy; (3) with incomplete clinical or follow-up information.

### TACE procedure

TACE procedures were performed by two interventional radiologists at each participating hospital, both of whom possessed at least 10 years of experience. Either conventional or drug-eluting beads TACE procedures, aiming for selective targeting of the tumor-feeding vessels, were standardized to reduce the heterogeneity and improve the embolization effect [20–22]. The decision to perform “on-demand” repeat TACE was based on the results of tumor markers and radiological examinations [23]. Details on the TACE procedure have been described in Supplementary materials (the “Treatment protocol” section). On-demand TACE was discontinued if any of the following conditions occurred: (1) Child-Pugh class C (uncontrollable ascites, severe jaundice, overt hepatic encephalopathy, or hepatorenal syndrome); (2) ECOG score > 2; or (3) continuous progression of target lesions after three TACE sessions according to the modified Response Evaluation Criteria in Solid Tumors‒mRECIST [24].

### Administrations of donafenib and ICIs

Donafenib was administered orally for the first time about 3 days after TACE at an initial dose of 200 mg twice daily. It was interrupted for about 3 days before and after “on-demand” TACE. The interval between the first use of Donafenib and ICIs was less than 1 week. All ICIs were administered based on the guidelines and availability in China, following their standard dose and frequency. Donafenib and ICIs treatment were continued until disease progression or unacceptable toxic effects. Details on agent administrations have been described in Supplementary materials (Supplement page 4 and Supplementary Table 1).

### Follow-up and assessments

All patients underwent follow-up assessments every 6–9 weeks after the initial treatment. Imaging examinations included but were not limited to enhanced abdominal MRI and chest-abdomen plain CT scans. Laboratory tests included but were not limited to tumor markers, complete blood cell count, comprehensive biochemical analysis, and coagulation panel. During each follow-up, tumor response was independently assessed by two radiologists or interventional physicians with over 5 years of experience at each center per the modified Response Evaluation Criteria in Solid Tumors‒mRECIST criteria. In the event of any discrepancies, a third senior radiologist with over 10 years of experience was consulted. Final decisions were reached through consensus discussion, taking into account serial imaging comparisons and complementary laboratory assessments, such as changes in tumor markers. All adverse events (AEs) were evaluated and recorded per the National Cancer Institute Common Terminology Criteria for Adverse Events‒CTCAE version 5.0 by the National Cancer Institute. The final follow-up date was November 30, 2024.

### Outcomes

The primary outcome was PFS. For both of the two groups, PFS was defined as the time from the initiation of TACE treatment to the first occurrence of tumor progression or death from any cause.

Secondary outcomes included OS rate (the proportion of patients alive at specified time points from the initiation of TACE treatment), ORR (the percentage of patients with confirmed complete or partial response), and safety.

### Statistical analysis

To minimize potential bias between the two groups, propensity score matching (PSM) analysis was performed using a 1:1 nearest-neighbor matching method without replacement with a caliper width of 0.1 times the standard deviation of the logit of the propensity score. The propensity scores were calculated using a logistic regression model. The variables were as follows: age, gender, hepatitis B virus‒HBV, cirrhosis, alanine aminotransferase‒ALT, aspartate aminotransferase‒AST, albumin‒ALB, total bilirubin‒TBIL, alpha-fetoprotein‒AFP, Child-Pugh grade, TACE type, and the up-to-seven criteria. Sensitivity analysis was conducted using different matching methods and factors to assess the robustness of the PSM analysis. Subgroup analysis was performed to identify potential risk factors related to PFS and OS after matching.

Sample size was calculated using PASS software (version 15.0; https://www.ncss.com/software/pass/). Continuous variables were summarized as mean ± standard deviation or median (interquartile range (IQR)), as appropriate. Categorical variables were summarized as frequencies with proportions. Continuous variables were analyzed using the Student *t*-test or Mann–Whitney *U* test, while categorical variables were analyzed using the χ^2^ test or Fisher’s exact test. The differences in PFS and OS between the two groups were compared using the log-rank test. Survival curves were plotted using the Kaplan–Meier method. Univariate and multivariate analyses for matched subgroups were performed using the Cox proportional hazards model, with results displayed in a forest plot.

A two-tailed *p*-value of < 0.05 was considered statistically significant. All statistical analyses were performed using SPSS (version 26.0; IBM) and R software (version 4.3.2; R Project for Statistical Computing, http://www.r-project.org).

## Results

### Patient characteristics

A total of 364 patients were included in this study, with 192 patients receiving TACE plus donafenib and ICIs, and 172 patients receiving TACE monotherapy. Before PSM, the combination therapy group showed significantly higher tumor burden, a greater proportion of conventional TACE treatments, and a higher rate of hepatitis B infection. After matching, 127 patients from each group remained in the analysis, totaling 254 patients with the baseline up-to-seven distribution (≤ 7 = 30%, > 7 = 70%). No significant differences in baseline characteristics were observed between the two groups (Table 1). To demonstrate the quality of matching, we have added the absolute standardized mean differences of propensity score matching (Supplementary Fig. 1). After matching, all covariates showed standardized mean difference values below the conventional threshold of 0.1, indicating adequate covariate balance between the two groups. To further illustrate the overlap of propensity scores, we provided density plots of propensity score distributions for the two groups, both before and after matching (Supplementary Fig. S2). The median follow-up time for the entire cohort was 20.0 months (interquartile range (IQR) 13.2‒30.9). After matching, the median follow-up time was 17.7 months (IQR 14.0‒33.7) for the combination therapy group, and 23.8 months (IQR 12.3‒27.6) for the monotherapy group. According to the reverse Kaplan–Meier analysis, after matching, the median follow-up time was 19.1 months (95% confidence interval (CI) 6.7‒21.5) for the combination therapy group and 32.2 months (95% CI 30.8‒33.7) for the monotherapy group.

**Table 1:** Patient baseline characteristics of combination and monotherapy groups before and after PSM

Characteristics	Before PSM			After PSM		
	TACE (*n* = 172)	TACE + D + I (*n* = 192)	*p*-value	TACE (*n* = 127)	TACE + D + I (*n* = 127)	*p*-value
Age, mean ± SD	59.99 ± 10.96	59.98 ± 9.96	0.993	59.90 ± 11.52	60.17 ± 10.70	0.844
Gender, *n* (%)			0.301			0.628
Male	139 (80.81)	163 (84.90)		102 (80.31)	105 (82.68)	
Female	33 (19.19)	29 (15.10)		25 (19.69)	22 (17.32)	
Child-Pugh, *n* (%)			0.787			0.616
A	146 (84.88)	161 (83.85)		107 (84.25)	104 (81.89)	
B	26 (15.12)	31 (16.15)		20 (15.75)	23 (18.11)	
Cirrhosis, *n* (%)			0.148			0.796
No	69 (40.12)	63 (32.81)		47 (37.01)	49 (38.58)	
Yes	103 (59.88)	129 (67.19)		80 (62.99)	78 (61.42)	
HBV, *n* (%)			< 0.001			1.000
No	50 (29.07)	27 (14.06)		25 (19.69)	25 (19.69)	
Yes	122 (70.93)	165 (85.94)		102 (80.31)	102 (80.31)	
Tacetype, *n* (%)			< 0.001			0.394
cTACE	118 (68.60)	171 (89.06)		104 (81.89)	109 (85.83)	
DEB-TACE	54 (31.40)	21 (10.94)		23 (18.11)	18 (14.17)	
ALT, *n* (%)			0.662			0.891
< 50 U/L	121 (70.35)	131 (68.23)		89 (70.08)	88 (69.29)	
≥ 50 U/L	51 (29.65)	61 (31.77)		38 (29.92)	39 (30.71)	
AST, *n* (%)			0.148			0.802
< 40 U/L	91 (52.91)	87 (45.31)		65 (51.18)	67 (52.76)	
≥ 40 U/L	81 (47.09)	105 (54.69)		62 (48.82)	60 (47.24)	
TBIL, *n* (%)			0.369			1.000
< 20 μmol/L	117 (68.02)	122 (63.54)		87 (68.50)	87 (68.50)	
≥ 20 μmol/L	55 (31.98)	70 (36.46)		40 (31.50)	40 (31.50)	
ALB, *n* (%)			0.052			0.258
< 40 g/L	81 (47.09)	110 (57.29)		64 (50.39)	55 (43.31)	
≥ 40 g/L	91 (52.91)	82 (42.71)		63 (49.61)	72 (56.69)	
AFP, *n* (%)			0.817			0.888
< 400 ng/mL	129 (75.00)	146 (76.04)		92 (72.44)	93 (73.23)	
≥ 400 ng/mL	43 (25.00)	46 (23.96)		35 (27.56)	34 (26.77)	
Up-to-seven criteria, *n* (%)			0.004			0.784
Within	61 (35.47)	42 (21.88)		39 (30.71)	37 (29.13)	
Beyond	111 (64.53)	150 (78.12)		88 (69.29)	90 (70.87)	

*AFP* Alpha-fetoprotein, *ALB* Albumin, *ALT* Alanine aminotransferase, *AST* Aspartate aminotransferase, *HBV* Hepatitis B virus, *ICIs* Immune checkpoint inhibitors, *SD* Standard deviation, *TACE* Transarterial chemoembolization, *TACE* + *D* + *I* TACE plus donafenib and ICIs, *TBIL* Total bilirubin

### Efficacy

Before PSM, the median PFS in the combination therapy group was 17.9 months (95% CI 14.1–21.6), significantly longer than the monotherapy group (15.3 months [95% CI 13.2–17.4]; hazard ratio (HR) 0.706 [95% CI 0.537‒0.927]; *p* = 0.012). The median OS was not reached in either group, but a significant difference was observed (HR 0.539 [95% CI 0.354‒0.821], *p* = 0.003). The 1-year OS rate was 94.8% (95% CI 91.5‒98.2) in the combination therapy group and 82.6% (95% CI 77.1‒88.4) in the monotherapy group, while the 2-year OS rate was 77.2% (95% CI 69.6‒85.6) *versus* 63.9% (95% CI 56.9‒71.7), respectively (Supplementary Fig. S3).

**Fig. 2:**
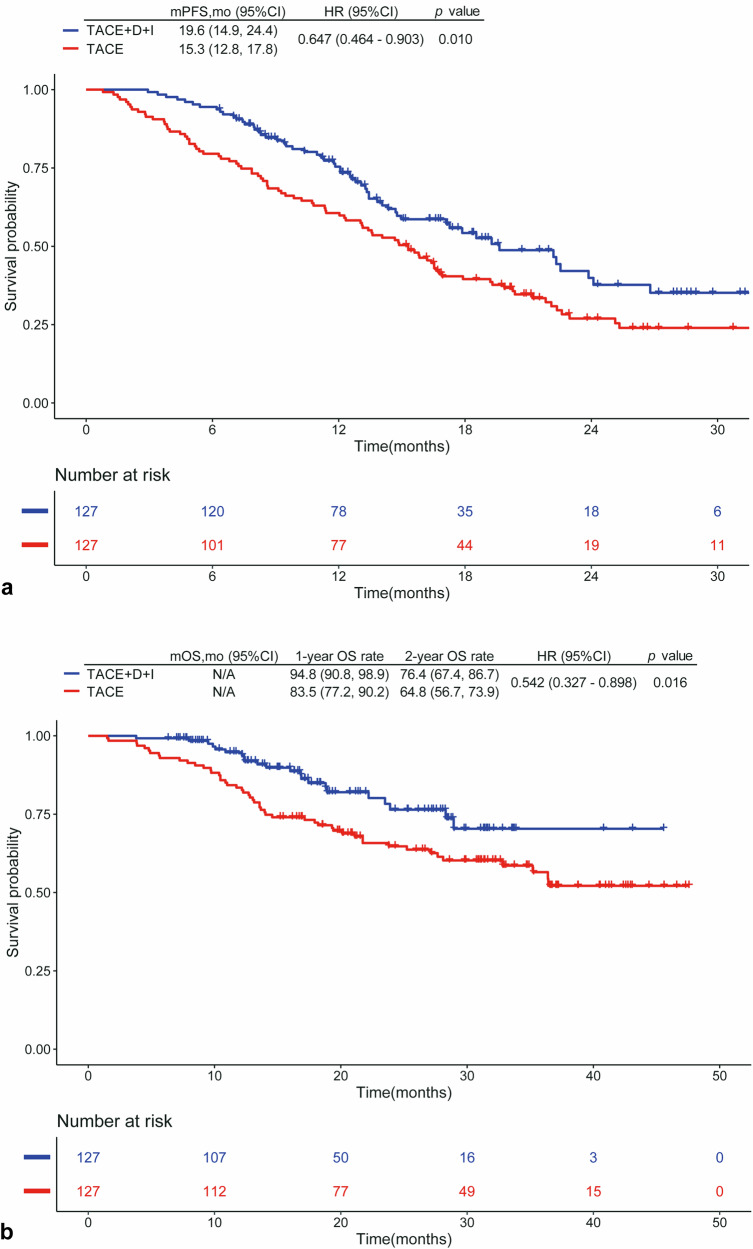
Kaplan–Meier analysis of progression-free survival (**a**) and overall survival (**b**) after matching

**Fig. 3:**
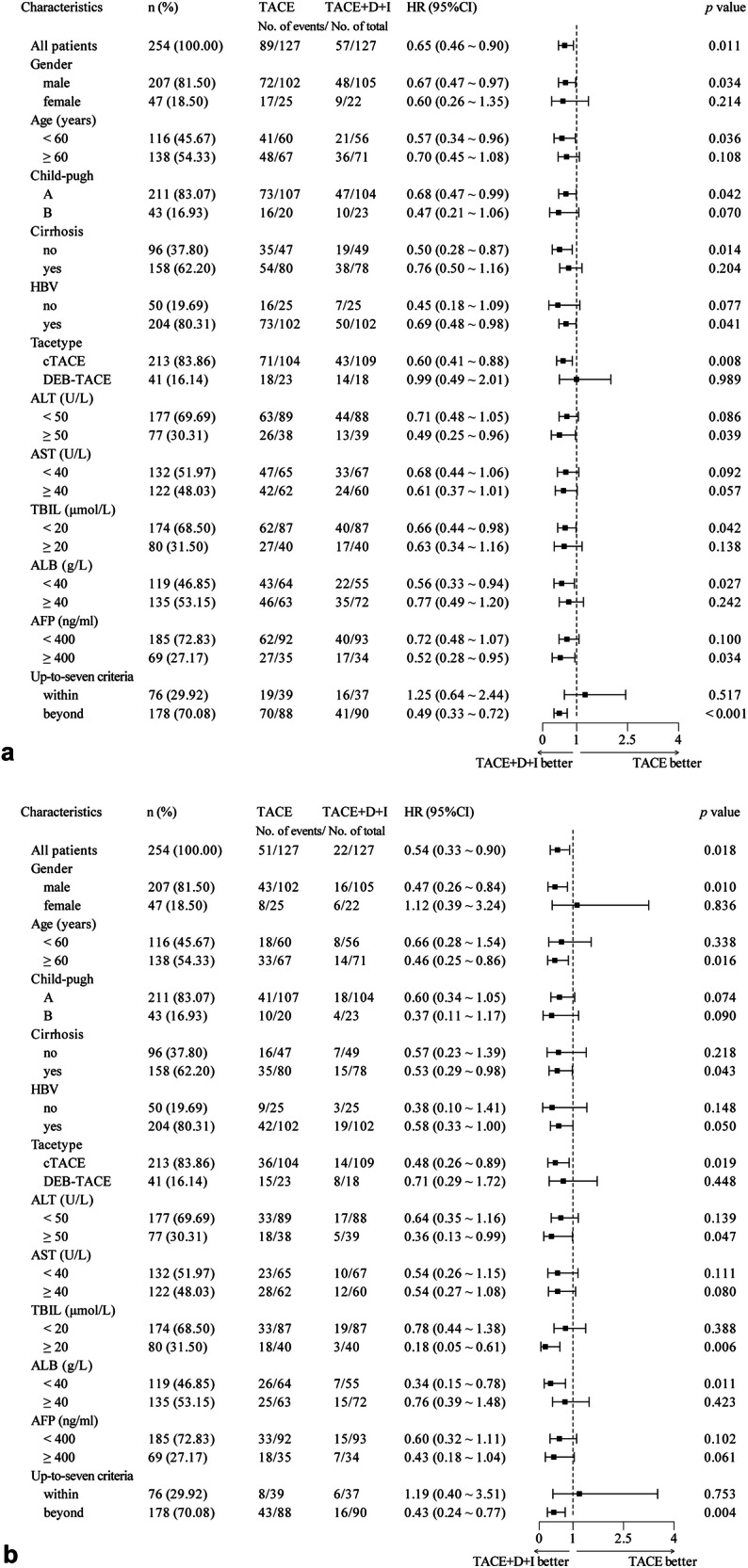
Forest plot for subgroup analysis of progression-free survival (**a**) and overall survival (**b**)

After matching, the median PFS in the combination therapy group was 19.6 months (95% CI 14.9‒24.4), significantly longer than in the monotherapy group (15.3 months [95% CI 12.8‒17.8]; HR 0.647 [95% CI 0.464‒0.903]; *p* = 0.010). The median OS was also not reached in either group, but a significant difference was observed (HR 0.542 [95% CI 0.327‒0.898], *p* = 0.016). The 1-year OS rate was 94.8% (95% CI 90.8‒98.9) in the combination therapy group and 83.5% (95% CI 77.2‒90.2) in the monotherapy group, while the 2-year OS rate was 76.4% (95% CI 67.4‒86.7) *versus* 64.8% (95% CI 56.7‒73.9), respectively (Fig. 2). The combination therapy group showed a significantly higher ORR compared with the TACE monotherapy group (78.9% *versus* 62.5%; *p* = 0.002). Multivariate Cox proportional hazards model showed that combination therapy was an independent positive prognostic factor for both PFS and OS in the matched cohort (for PFS, HR 0.601 [95% CI 0.428‒0.844], *p* = 0.003; for OS, HR 0.538 [95% CI 0.323‒0.897], *p* = 0.017) (Table 2). For the inverse probability of treatment weighting (IPTW) analysis, multivariate Cox regression also showed that the combination therapy was an independent prognostic factor for both PFS (HR = 0.725, 95% CI 0.542–0.971, *p* = 0.031) and OS (HR = 0.589, 95% CI 0.381–0.910, *p* = 0.017) in the weighted cohort. The sensitivity analysis supported that these average treatment effect (ATE)-oriented estimates are consistent with the average treatment effect on the treated (ATT)-oriented results obtained from PSM. Subgroup analysis indicated that, compared to the monotherapy group, the combination therapy group showed a consistent trend toward better PFS and OS benefits (Fig. 3).

**Table 2:** Univariate and multivariate analyses of factors associated with progression-free survival and overall survival after matching

Characteristics	Univariable		Multivariable	
	HR (95% CI)	*p*-value	HR (95% CI)	*p*-value
PFS analyses				
Age (> 60 *versus* ≤ 60)	1.009 (0.989–1.031)	0.379		
Gender (female *versus* male)	1.045 (0.682–1.600)	0.840		
Cirrhosis (yes *versus* no)	1.076 (0.769–1.506)	0.669		
HBV (yes *versus* no)	1.261 (0.807–1.968)	0.308		
ALT (> 50 *versus* ≤ 50)	0.842 (0.583–1.215)	0.358		
AST (> 40 *versus* ≤ 40)	0.936 (0.676–1.297)	0.692		
TBIL (> 20 *versus* ≤ 20)	0.937 (0.658–1.335)	0.720		
ALB (> 40 *versus* ≤ 40)	1.104 (0.796–1.531)	0.553		
AFP (> 400 *versus* ≤ 400)	1.330 (0.934–1.896)	0.114		
TACE type (DEB-TACE *versus* cTACE)	1.946 (1.312–2.885)	< 0.001	1.770 (1.162–2.695)	0.008
Child-Pugh (B *versus* A)	1.124 (0.735–1.717)	0.591		
Up-to-seven criteria (> 7 *versus* ≤ 7)	1.682 (1.150–2.462)	0.007	1.870 (1.241–2.817)	0.003
Treatment (TACE + D + I *versus* TACE)	0.647 (0.464–0.903)	0.010	0.601 (0.428–0.844)	0.003
OS analyses				
Age (> 60 *versus* ≤ 60)	1.407 (0.948–2.088)	0.090		
Gender (female *versus* male)	1.157 (0.645–2.073)	0.625		
Cirrhosis (yes *versus* no)	1.484 (0.905–2.433)	0.118		
HBV (yes *versus* no)	1.086 (0.584–2.019)	0.794		
ALT (> 50 *versus* ≤ 50)	1.197 (0.729–1.965)	0.477		
AST (> 40 *versus* ≤ 40)	1.502 (0.947–2.384)	0.084		
TBIL (> 20 *versus* ≤ 20)	0.916 (0.551–1.521)	0.733		
ALB (> 40 *versus* ≤ 40)	1.064 (0.671–1.688)	0.791		
AFP (> 400 *versus* ≤ 400)	1.394 (0.859–2.260)	0.179		
TACE type (DEB-TACE *versus* cTACE)	2.619 (1.598–4.295)	< 0.001	3.242 (1.857–5.662)	< 0.001
Child-Pugh (B *versus* A)	1.284 (0.717–2.301)	0.400		
Up-to-seven criteria (> 7 *versus* ≤ 7)	1.900 (1.060–3.403)	0.031	1.843 (1.008–3.369)	0.047
Treatment (TACE + D + I *versus* TACE)	0.542 (0.327–0.898)	0.016	0.538 (0.323–0.897)	0.017

*AFP* Alpha-fetoprotein, *ALB* Albumin, *ALT* Alanine aminotransferase, *AST* Aspartate aminotransferase, *HBV* Hepatitis B virus, *HR* Hazard ratio, *TACE* Transarterial chemoembolization, *TACE* + *D* + *I* TACE plus donafenib and ICIs, *TBIL* Total bilirubin

Subgroup analysis based on baseline tumor burden showed that for the subgroup beyond the up-to-seven criteria, the combination therapy group demonstrated better PFS (19.3 months [95% CI 14.7‒23.8] *versus* 11.4 months [95% CI 8.0‒14.8], in the monotherapy group; HR 0.491 [95% CI 0.334‒0.722], *p* < 0.001). However, for the subgroup within the up-to-seven criteria, no statistically significant difference in PFS was observed between the two groups (22.3 months [95% CI 11.5‒33.2] *versus* 21.8 months [95% CI 19.0‒24.7], respectively; HR 0.801 [95% CI 0.410‒1.567], *p* = 0.517) (Supplementary Fig. S4 and Table S2).

To evaluate whether the efficacy of the combination therapy varied by tumor burden, interaction term was included between treatment modality and up-to-seven criteria in multivariable Cox regression models. As shown in Table 3, the interaction term was statistically significant for both PFS (HR = 0.684, 95% CI 0.478‒0.979, *p* = 0.038) and OS (HR = 0.569, 95% CI 0.326‒0.994, *p* = 0.047), indicating a differential treatment effect across tumor burden strata.

**Table 3:** Interaction analysis (treatment × up-to-seven) after matching

Characteristics	β	SE	*z* value	HR (95% CI)	*p*-value
PFS analyses					
Up-to-seven criteria (> 7 *versus* ≤ 7)	0.520	0.194	2.678	1.682 (1.150–2.462)	0.007
Treatment (TACE + D + I *versus* TACE)	-0.435	0.170	-2.556	0.647 (0.464–0.903)	0.010
Interaction term (treatment × up-to-seven)	-0.380	0.183	-2.077	0.684 (0.478–0.979)	0.038
OS analyses					
Up-to-seven criteria (> 7 *versus* ≤ 7)	0.642	0.297	2.157	1.900 (1.060–3.403)	0.031
Treatment (TACE + D + I *versus* TACE)	-0.612	0.258	-2.374	0.542 (0.327–0.898)	0.016
Interaction term (treatment × up-to-seven)	-0.564	0.284	-1.982	0.569 (0.326–0.994)	0.047

*OS* Overall survival, *PFS* Progression-free survival, *TACE* Transarterial chemoembolization plus donafenib and immune checkpoint inhibitors, *SE* Standard error

The prognostic impact of different types of ICIs used in the combination therapy group was further explored and was not associated with survival outcomes in univariable Cox regression analysis (Supplementary Table S3).

### Safety

Regarding TACE-related AEs, after matching, the combination therapy group had 70 cases (55.1%) of grade-1/2 AEs and 5 cases (3.9%) of grade-3/4 AEs. The monotherapy group had 86 cases (67.7%) of grade-1/2 AEs and 7 cases (5.5%) of grade-3/4 AEs. No grade 5 AEs were observed in either group, and no treatment-related fatalities occurred during the study (Table 4). In the combination therapy group, any adverse event (≥ 10%) included hepatic impairment (44.1%), abdominal pain (20.5%), gastrointestinal reactions (22.8%), and pyrexia (14.2%) (Supplementary Table 4). In the monotherapy group, any adverse event (≥ 10%) included hepatic impairment (67.7%), abdominal pain (34.6%), gastrointestinal reactions (15.0%), and pyrexia (19.7%) (Supplementary Table S5).

**Table 4:** Adverse events of the two groups after matching

Types	TACE (*n* = 127)	TACE + D + I (*n* = 127)
TACE-related AEs		
Grade-1/2, *n* (%)	86 (67.7%)	70 (55.1%)
Grade-3/4, *n* (%)	7 (5.5%)	5 (3.9%)
ICIs-related AEs		
Grade-1/2, *n* (%)	-	45 (35.4%)
Grade-3/4, *n* (%)	-	4 (3.1%)
Donafenib-related AEs		
Grade-1/2, *n* (%)	-	65 (51.2%)
Grade-3/4, *n* (%)	-	7 (5.5%)

*AEs* Adverse events, *ICIs* Immune checkpoint inhibitors, *TACE* Transarterial chemoembolization, *TACE* + *D* + *I* TACE plus donafenib and ICIs

Regarding donafenib-related AEs, after matching, the combination therapy group had 65 cases (51.2%) of grade-1/2 AEs and 7 cases (5.5%) of grade-3/4 AEs (Table 4). Any adverse event (≥ 10%) included skin toxicity (39.4%), hypertension (11.8%), and gastrointestinal reactions (10.2%) (Supplementary Table S4).

Regarding ICIs-related AEs, after matching, the combination therapy group had 45 cases (35.4%) of grade-1/2 AEs and 4 cases (3.1%) of grade-3/4 AEs (Table 4). No adverse event at any grade exceeded 10% (Supplementary Table S4).

In the combination therapy group, 3 patients (2.4%) discontinued ICIs and 6 patients (4.7%) discontinued donafenib due to AEs, while 12 patients (9.4%) had their donafenib dose reduced.

## Discussion

This nationwide, multicenter, retrospective matched cohort study demonstrated that compared to TACE monotherapy, the combination therapy of TACE plus donafenib and ICIs significantly improved PFS in intermediate HCC (19.6 months [95% CI 14.9‒24.4] *versus* 15.3 months [95% CI 12.8‒17.8], HR 0.647 [95% CI 0.464‒0.903], *p* = 0.010), with higher OS rates (94.8% *versus* 83.5%, 1-year OS rate; 76.4% *versus* 64.8%, 2-year OS rate; HR 0.542 [95% CI 0.327‒0.898], *p* = 0.016) and ORR (78.9% *versus* 62.5%, *p* = 0.002). Grade-3/4 AEs occurred more frequently in the combination therapy group (12.5%) compared to the TACE monotherapy group (5.5%). Because 30% of patients were excluded by PSM, our estimates primarily reflect the average treatment effect on the treated in the matched population. Unmatched patients differed mainly in TACE type and ALB level (Supplementary Table 6), so caution is warranted when generalizing to those subgroups.

For unresectable HCC, the efficacy of combination of TACE plus PD-(L)1 inhibitors and molecular targeted therapies has been well established, as exemplified by the CHANCE001 real-world study, which demonstrated that the combination therapy significantly improved both PFS (mPFS: 9.5 months *versus* 8.0 months, HR 0.70, *p* = 0.002) and OS (mOS: 19.2 months *versus* 15.7 months, HR 0.63, *p* = 0.001) [11]. For the specific combination in patients with intermediate HCC, such as lenvatinib and pembrolizumab, LEAP012 also demonstrated comparable efficacy outcomes [9]. The latest data confirmed the median PFS in the combination therapy group extended from 10.0 months to 14.6 months (HR 0.66, *p* < 0.001) compared to TACE monotherapy [25]. Donafenib has been evaluated in clinical trials assessing combination therapy as well [18, 19, 26]. A phase I clinical trial evaluated the preliminary efficacy of donafenib in combination with TACE and PD-(L)1 inhibitors for HCC [27]. The study demonstrated a total ORR of 62.5%. Notably, the ORR for patients with intermediate HCC was significantly higher, reaching 83.3%. These results were consistent with those observed in our study. Our study showed even longer PFS and OS. Because, for intermediate HCC, the combination therapy with donafenib was taken, while the control cohort received the standard first-line treatment. This could also be attributed to the better liver function, good performance status, and the widespread use of superselective chemoembolization in the patients of our cohort [28]. Additionally, the later enrollment period, along with increasingly standardized surgical techniques, treatment protocols, and enhanced postoperative management, may also contribute to the favorable prognosis observed in our study [22, 29, 30].

The efficacy benefits of TACE plus donafenib and ICIs were consistent across clinical subgroups, including those related to HCC (liver function status, hepatitis B virus etiology, cirrhosis, baseline level of tumor markers). However, in patients within up-to-seven criteria, numerical trends favored combination therapy, but differences did not reach statistical significance, likely owing to fewer events and good outcomes in both arms. The interaction term suggests that the relative benefit of combination therapy is greater in patients beyond the up-to-seven criteria, whereas treatment for low-burden cases should be individualized. The advantages of TKIs and ICIs were typically observed in patients with higher tumor burden or those at increased risk of recurrence [31–33]. The multivariate Cox regression and subgroup analyses in our study corroborated these findings. In patients with low tumor burden, the additional benefits of TKIs and ICIs may be less pronounced, particularly when liver function is well-preserved, tumors are localized, and TACE achieves optimal control [34, 35]. Nevertheless, the combination therapy may still offer significant survival benefits in patients with low tumor burden [36, 37]. The TACTICS trial demonstrated that combination therapy was more favorable regardless of baseline tumor burden, with a hazard ratio (HR) of 0.48 (95% CI 0.24–0.94) for patients beyond the up-to-seven criteria and 0.64 (95% CI 0.27–1.04) for those within the criteria [38]. The EMERALD-1 trial demonstrated that patients, regardless of baseline tumor burden, derive benefit from the combination therapy [10]. Overall, these findings support the use of combination therapy as a first-line option for patients beyond the up-to-seven criteria, while treatment for low-burden HCC should be individualized.

The increase in the incidence of AEs is expected, given the addition of donafenib and ICIs, both of which are known to contribute to systemic toxicities. Specifically, TACE-related grade-3/4 AEs were comparable between the two groups (3.9% in the combination group *versus* 5.5% in the monotherapy group), suggesting that TACE alone was not responsible for the increased toxicity. These findings are consistent with prior literature. EMERALD-1 showed that the incidence of any treatment-related AEs was 80.5% and 45.0% in the D + B + TACE and TACE monotherapy groups, respectively, with TACE-related AEs occurring at rates of 50.6% and 47.5% [10]. Similarly, the CHANCE001 study reported that grade-3/4 AEs related to ICIs and targeted therapies occurred in 5.7% and 9.2% of patients, respectively, while TACE-related grade-3/4 AEs were 4.3% in the combination group *versus* 6.1% in the monotherapy group [11]. The addition of ICIs slightly increased the incidence of AEs in the combination of TACE and donafenib [19]. Moreover, Li et al reported 11.3% grade-3 treatment-related AEs in a similar combination cohort [18]. These data further support that the observed toxicity profile in this study aligns with previously established safety benchmarks. Taken together, the inclusion of donafenib (5.5%) and ICIs (3.1%) contributed to the additive burden, which still remains within acceptable and manageable limits.

The type of TACE technique was indeed considered in our statistical analysis. As reported in the main text, TACE type was identified as an independent prognostic factor influencing survival outcomes. However, previous literature has shown that conventional and drug-eluting beads TACE result in broadly similar clinical benefits for HCC [39, 40]. Thus, while we acknowledge the prognostic significance of TACE type, we did not overemphasize it in the discussion. We have ensured that baseline characteristics by TACE type and their outcome associations are clearly presented.

This study has several limitations. First, although this nationwide, multicenter, retrospective study used PSM to minimize bias, residual confounding or inherent selection biases associated with retrospective study designs cannot be fully eliminated. While this approach, particularly with a narrow caliper, improves covariate balance and internal validity, it may limit the generalizability. Unmeasured variables, such as subtle differences in tumor biology, physician decision-making, or patient comorbidities, may still have influenced treatment allocation and outcomes. Therefore, this study’s findings should be interpreted with caution and validated in prospective randomized controlled trials. Second, there was a discrepancy in the enrollment timing, where some patients in the TACE monotherapy group were enrolled earlier than those in the combination therapy group. This situation led to a shorter median follow-up time in the combination therapy group. However, this is consistent with real-world clinical practice. Donafenib was officially launched in China in June 2021. With the continuous development and incorporation of ICIs and TKIs as first-line treatments for patients with intermediate-advanced HCC, the proportion of TACE monotherapy in clinical practice has gradually decreased, with increasing use of combination therapies involving systemic treatments. Such discrepancies may introduce bias and affect the interpretability of survival-related outcomes. Specifically, patients in the combination group had less time for long-term outcomes such as OS or late adverse events to manifest. As a result, comparisons of efficacy or safety between the two groups may be influenced by differences in follow-up opportunity rather than actual therapeutic effect. Moreover, this time-related imbalance may also limit the generalizability of our findings to broader patient populations or healthcare settings. In particular, centers or regions that adopted systemic therapies earlier or later may observe different patterns of treatment efficacy and tolerability. Third, the kinds of ICIs used in this study were not uniform. After progression, most patients in the combination group received second-line TKIs or ICIs, whereas patients initially treated with TACE monotherapy predominantly started first-line systemic therapy. Specific regimens varied across centers and could not be reliably tabulated. The optimal combinations of donafenib with specific ICIs can be explored in future research with larger sample sizes.

While current guidelines recommend TACE as standard treatment for intermediate HCC, they also acknowledge the potential role of systemic therapies, particularly in patients with high tumor burden or poor response to TACE monotherapy. This study supports this shift and provides further justification for incorporating TACE-based combination regimens, especially with donafenib, into routine practice. These findings may help guide individualized treatment decisions and inform future updates of regional treatment guidelines.

## Supplementary information

**Additional file 1: Table S1.** Agents administration protocol. **Table S2**. Subgroup analysis based on baseline tumor burden. **Table S3.** The exact composition and distribution of ICIs and univariate Cox analysis associated with progression-free survival and overall survival. **Table S4.** Adverse events in combination group after matching. **Table S5.** Adverse events in monotherapy group after matching. **Table S6.** The baseline characteristics of the unmatched patients. **Fig. S1.** Absolute standardized mean difference of propensity score matching. **Fig. S2.** Density Plots before and after propensity score matching. **Fig. S3.** Kaplan–Meier analysis of progression-free survival (**a**) and overall survival (**b**) before matching. **Fig. S4.** Kaplan–Meier analysis of progression-free survival for subgroup analysis based on baseline tumor burden after matching. a for the subgroup beyond the up-to-seven criteria. **b** for the subgroup within the up-to-seven criteria. **Fig. S5.** Kaplan–Meier analysis of progression-free survival for sensitivity analysis. **a** for matching at a ratio of 2:1. **b** for matching with several key clinical factors.

## Data Availability

The datasets used and/or analyzed during the current study are available from the corresponding authors on reasonable request.

## Abbreviations

AEs: Adverse events
BCLC: Barcelona Clinic Liver Cancer
CI: Confidence interval
ECOG: Eastern Cooperative Oncology Group
HCC: Hepatocellular carcinoma
HR: Hazard ratio
ICIs: Immune checkpoint inhibitors
IQR: Interquartile range
ORR: Objective response rate
OS: Overall survival
PFS: Progression-free survival
PSM: Propensity score matching
TACE: Transarterial chemoembolization
TKIs: Tyrosine kinase inhibitors
