# Supplementary material for: TACE plus donafenib and immune checkpoint inhibitors for intermediate HCC (CHANCE2410 study): a propensity score matching analysis
Source: Eur Radiol Exp. 2026 Jun 16;10:88. doi: 10.1186/s41747-026-00748-5 (PMC13272723; doi:10.1186/s41747-026-00748-5)
Supplement: Supplementary file 1 — Additional file 1: Table S1. Agents administration protocol. Table S2. Subgroup analysis based on baseline tumor burden. Table S3. The exact composition and distribution of ICIs and univariate Cox analysis associated with progression-free survival and overall survival. Table S4. Adverse events in combination group after matching. Table S5. Adverse events in monotherapy group after matching. Table S6. The baseline characteristics of the unmatched patients. Fig. S1. Absolute standardized mean difference of propensity score matching. Fig. S2. Density Plots before and after propensity score matching. Fig. S3. Kaplan–Meier analysis of progression-free survival (a) and overall survival (b) before matching. Fig. S4. Kaplan–Meier analysis of progression-free survival for subgroup analysis based on baseline tumor burden after matching. a for the subgroup beyond the up-to-seven criteria. b for the subgroup within the up-to-seven criteria. Fig. S5. Kaplan–Meier analysis of progression-free survival for sensitivity analysis. a for matching at a ratio of 2:1. b for matching with several key clinical factors. [file 41747_2026_748_MOESM1_ESM.pdf]

# TACE plus donafenib and immune checkpoint inhibitors for intermediate HCC (CHANCE2410 study): a propensity score matching analysis

## ELECTRONIC SUPPLEMENTARY MATERIAL

**This PDF file includes:**


Materials and Methods

Results

Supplementary Tables S1 to S6

Supplementary Figures S1 to S5

References

the list of CHANCE2410 investigators

## Materials and Methods

### Treatment protocol

### Transarterial chemoembolization procedure

Patients in this study received either conventional transarterial chemoembolization (cTACE) or drug-eluting beads TACE (DEB-TACE). All procedures were carried out in accordance with standardized protocols across all participating centers[1, 2]. All the TACE procedures were performed by physicians with at least 10 years of experience on interventional radiology from participating centers. During the procedure, it was essential to achieve clear visualization of all tumor-feeding arteries, including vessels' origin, variant anatomy, and the presence of collateral or ectopic blood supply. Both cTACE and DEB-TACE aimed for selective targeting of the tumor-feeding vessels to enhance treatment efficacy while minimizing associated complications. In cases of non-selective TACE, the procedural endpoint was characterized by the "tree in winter" appearance.

For cTACE, an emulsion of mixtures of lipiodol (2–20 ml) and chemotherapeutic drugs was injected into the tumor-feeding arteries. Doxorubicin is the most common single chemotherapeutic drug. The dosage of the chemotherapeutic agent was determined based on factors such as body surface area, liver function, weight, or according to empirical guidelines. Chemotherapeutic drugs including doxorubicin (10–100 mg), epirubicin (5–120 mg), oxaliplatin (100–200 mg), cisplatin (10–100 mg) and other drugs were selected according to clinical practice of the participating centers. The ethiodized oil and chemotherapeutic agents should be thoroughly mixed to form a stable emulsion, specifically configured as a "water-in-oil" emulsifier to enhance its stability. The volume ratio of ethiodized oil to the drug aqueous solution is typically 2:1. The volume of ethiodized oil administered is generally determined based on the size and vascularity of the tumor, with common dosages ranging from 5 to 15 mL. Finally, following the administration of the ethiodized oil chemoembolic emulsion, particulate embolic agents such as standardized gelatin sponge particles, microspheres, or polyvinyl alcohol particles should be used to achieve an optimal embolization endpoint.

For DEB-TACE, a dose of 2–4 mL of drug-eluting beads, including DC beads (Biocompatibles, Farnham, United Kingdom), Callispheres beads (Jiangsu Hengrui Medicine Co., Ltd., Jiangsu, China), or HepaSpheres beads (Biosphere Medical, Inc., South Jordan, UT), with diameters of 100–300 μm or 300–500 μm, was loaded with epirubicin (up to a maximum dose of 100 mg) and injected into the tumor-feeding arteries. If the desired embolization endpoint was not achieved, additional embolization was performed.

"On demand" TACE procedures were repeated based on the demonstration of viable tumors or intrahepatic recurrences by contrast-enhanced computed tomography (CT) or magnetic resonance imaging (MRI)[3]. TACE was repeated when residual viable tumors were confirmed or new lesions developed in patients with adequate liver function.

TACE was discontinued if one of the following conditions occurred: (1) deterioration of liver function to Child-Pugh C (uncontrollable ascites, severe jaundice, overt hepatic encephalopathy, or hepatorenal syndrome); (2) Eastern Cooperative Oncology Group (ECOG > 2); (3) continued progression of target lesions after three TACE sessions according to the modified Response Evaluation Criteria in Solid Tumors (mRECIST).

**Administration of Donafenib**

Donafenib was administered orally 3 days after TACE at an initial dose of 200 mg twice daily. Dose reduction or interruption of donafenib due to adverse events was acceptable. If donafenib-related adverse reactions could be tolerated, the dose was adjusted to 200 mg once daily. Patients continued to receive donafenib until either disease progression or unacceptable toxicities occurred.

**Administration of immune checkpoint inhibitors**

A range of immune checkpoint inhibitors (ICIs), such as atezolizumab, camrelizumab, sintilimab, toripalimab and tislelizumab were utilized, in accordance with guidelines and availability in China (Supplementary Table S1). The administration of these ICIs adhered to prescribed dosages and frequencies. While dose reduction was not permitted, interruption of ICIs due to adverse events was acceptable. Patients continued to receive ICIs until either disease progression or unacceptable toxicities occurred.

**Post-progression therapy**

In the combination therapy group, patients typically switched to second-line TKIs or ICIs, depending on individual clinical status. In the TACE monotherapy group, patients who progressed commonly received first-line systemic therapy as the subsequent treatment, in accordance with standard treatment guidelines.

**Sensitivity analyses**

The propensity score matching analysis was performed using a 2:1 nearest-neighbor method without replacement and a caliper width of 0.1 times the standard deviation of the logit of the propensity score. The propensity scores were calculated using a logistic regression model. The variables were as follows: age, gender, hepatitis B virus (HBV), cirrhosis, alanine aminotransferase (ALT), aspartate aminotransferase (AST), albumin (ALB), total bilirubin (TBIL), alpha-fetoprotein (AFP), Child-Pugh grade, TACE type, and the up-to-seven criteria. After matching, we conducted a Kaplan–Meier curve analysis and an adjusted Cox proportional hazards model incorporating the above covariates.

Then, we performed propensity score matching with several key clinical factors (HBV, TACE type, and the up-to-seven criteria), which indicated patients' etiology, interventional treatment method, and tumor burden, not balanced before matching. The 1:1 nearest-neighbor method without replacement with caliper widths of 0.1 times the standard deviation of the logit of the propensity score was used.

For the inverse probability of treatment weighting analysis, we calculated the probability of receiving the combination therapy (propensity score) for each patient using a logistic regression model. The model included the following variables: age, gender, HBV, cirrhosis, ALT, AST, ALB, TBIL, AFP, Child-Pugh grade, TACE type, and the up-to-seven criteria. We calculated individual weights using the propensity score as follows: 1/propensity score for patients receiving the combination therapy, and 1/ (1–propensity score) for monotherapy.

## Results

### Sample size calculation

As supported by AASLD Consensus Conferencepreviously and previously reported data, the median PFS for TACE monotherapy was set as 8 months while the median PFS for combination group was set as 13 months[4, 5]. Follow-up time was set as 6 months. The proportion dropping out of each group was 0.05 per year. A two-sided log-rank test with an overall sample size of 230 patients (115 in each group) achieves 80% power at a 0.05 significance level. Above sample size calculation were performed using PASS (version 15.0).

### Sensitivity analyses

After 1:1 PSM, 127 patients were retained in each group, resulting in a sample loss of approximately 30.2%. Compared with the matched cohort, the unmatched patients differed significantly only in TACE type ($p$ = 0.001) and ALB level ($p$ = 0.001), but were otherwise broadly comparable (Supplementary Table S6).

After matching at a ratio of 2:1, there was no difference of baseline characteristics between the two groups. After matching, 127 patients remained in the combination therapy group and 149 patients remained in the monotherapy group. The median PFS was 19.6 months (95% CI 15.0-26.8) in the combination therapy group, compared to 14.9 months (95% CI, 12.3-19.6) in the monotherapy group (hazard ratio [HR] 0.617 [95% CI 0.447-0.850], $p$ = 0.003). (Supplementary Fig. S5a)

After matching with several key clinical factors, the median PFS was 17.2 months (95% CI 14.1-22.0) in the combination therapy group, compared to 14.9 months (95% CI 12.3-16.9) in the monotherapy group (HR 0.706 [95% CI 0.521-0.956], $p$ = 0.024). (Supplementary Fig. S5b)

After adjusted the covariates, multivariate Cox regression analysis showed that combination therapy (for PFS, HR 0.725, 95% CI 0.542–0.971, $p$ = 0.031; for OS, HR 0.589, 95% CI 0.381–0.910, $p$ = 0.017) was the independent prognostic indicators in all patients weighting analysis cohort.

**Heterogeneous regimens**

The prognostic impact of different types of ICIs used in the combination therapy group was further explored (Supplementary Table S3). Before PSM, a total of 192 patients received ICIs, with tislelizumab (n=89) as the reference agent, followed by sintilimab (n=55), camrelizumab (n=39), toripalimab (n=5) and atezolizumab (n=4). Univariable Cox regression analysis showed no statistically significant difference in PFS among the different ICIs. Compared to the reference group (tislelizumab), the HRs for PFS were 0.701 (95% CI 0.425-1.156; $p = 0.164$) for sintilimab, 0.870 (95% CI 0.505-1.499; $p = 0.615$) for camrelizumab, 1.800 (95% CI 0.646-5.010; $p = 0.261$) for toripalimab and 0.000 (95% CI 0.000-Inf; $p = 0.994$) for atezolizumab. Similarly, the type of ICIs was not an independent risk factor affecting OS. The HRs for OS compared to tislelizumab were 0.708 (95% CI 0.308-1.628; $p = 0.416$) for sintilimab, 0.958 (95% CI 0.400-2.295; $p = 0.924$) for camrelizumab, 0.000 (95% CI 0.000-Inf; $p = 0.997$) for toripalimab and 0.000 (95% CI 0.000-Inf; $p = 0.999$) for atezolizumab.

After PSM, a total of 127 patients received ICIs, with tislelizumab (n=56) as the reference agent, followed by sintilimab (n=28), camrelizumab (n=38), toripalimab (n=3) and atezolizumab (n=2). Univariable Cox regression analysis showed no statistically significant difference in PFS among the different ICIs. Compared to the reference group (tislelizumab), the HRs for PFS were 0.652 (95% CI 0.342-1.242; $p = 0.193$) for sintilimab, 0.853 (95% CI 0.440-1.654; $p = 0.639$) for camrelizumab, 1.317 (95% CI 0.311-5.570; $p = 0.708$) for toripalimab and 0.000 (95% CI 0.000-Inf; $p = 0.996$) for atezolizumab. Similarly, the type of ICIs was not an independent risk factor affecting OS. The HRs for OS compared to tislelizumab were 0.852 (95% CI 0.315-2.304; $p = 0.416$) for sintilimab, 0.866 (95% CI 0.300-2.496; $p = 0.790$) for camrelizumab, 0.000 (95% CI 0.000-Inf; $p = 0.998$) for toripalimab and 0.000 (95% CI 0.000-Inf; $p = 1.000$) for atezolizumab.

**Supplementary Table S1.** Agents administration protocol.

| Agents | Targets | Administration |
|---|---|---|
| Atezolizumab, Tecentriq®, F. Hoffmann-La Roche AG, Basel, Switzerland | PD-L1 | 1200 mg once every 3 weeks, intravenous infusion |
| Sintilimab, Tyvyt®, Innovent Biologics, Inc., Suzhou, China | PD-1 | 200 mg once every 3 weeks, intravenous infusion |
| Camrelizumab, AiRuiKa®, Jiangsu Hengrui Medicine Co. Ltd, Suzhou, China | | 200 mg once or 3mg/kg once every 3 weeks, intravenous infusion |
| Tislelizumab, Baize'an®, BeiGene Ltd., Beijing, China | | 200 mg once every 3 weeks, intravenous infusion |
| Toripalimab, Tuoyi®, Junshi Bioscience Co., Ltd, Shanghai, China | | 240 mg once every 3 weeks, intravenous infusion |

**Supplementary Table S2.** Subgroup analysis based on baseline tumor burden.

| | subgroup beyond the up-to-seven criteria | subgroup within the up-to-seven criteria |
|---|---|---|
| mOS,month | NA | NA |
| TACE+D+I | NA | NA |
| TACE | 36.4 (26.1, 46.7) | NA |
| mPFS,month | 14.8 (12.4, 17.1) | 21.8 (19.3, 24.3) |
| TACE+D+I | 19.3 (14.7, 23.8) | 22.3 (11.5, 33.2) |
| TACE | 11.3 (8.0, 14.8) | 21.8 (19.0, 24.7) |
| ORR | 52.8% (94/178) | 52.6% (40/76) |
| TACE+D+I | 78.9% (71/90) | 78.4% (29/37) |
| TACE | 26.1% (23/88) | 28.2% (11/39) |
| any grade AE rates | 84.8% (151/178) | 84.2% (64/76) |
| TACE+D+I | 88.9% (80/90) | 86.5% (32/37) |
| TACE | 80.7% (71/88) | 82.1% (32/39) |

**Supplementary Table S3.** The exact composition and distribution of ICIs and univariate Cox analysis associated with progression-free survival and overall survival.

| Characteristics | n | PFS analyses | | OS analyses | |
|---|---|---|---|---|---|
| | | HR (95% CI) | *p* value | HR (95% CI) | *p* value |
| *Before PSM* | | | | | |
| ICIs type | 192 | | | | |
| tislelizumab | 89 | 1.000 (Reference) | | 1.000 (Reference) | |
| atezolizumab | 4 | 0.000 (0.000 ~ Inf) | 0.994 | 0.000 (0.000 ~ Inf) | 0.999 |
| camrelizumab | 39 | 0.870 (0.505 ~ 1.499) | 0.615 | 0.958 (0.400 ~ 2.295) | 0.924 |
| sintilimab | 55 | 0.701 (0.425 ~ 1.156) | 0.164 | 0.708 (0.308 ~ 1.628) | 0.416 |
| toripalimab | 5 | 1.800 (0.646 ~ 5.010) | 0.261 | 0.000 (0.000 ~ Inf) | 0.997 |
| *After PSM* | | | | | |
| ICIs type | 127 | | | | |
| tislelizumab | 56 | 1.000 (Reference) | | 1.000 (Reference) | |
| atezolizumab | 2 | 0.000 (0.000 ~ Inf) | 0.996 | 0.000 (0.000 ~ Inf) | 1.000 |
| camrelizumab | 38 | 0.853 (0.440 ~ 1.654) | 0.639 | 0.866 (0.300 ~ 2.496) | 0.790 |
| sintilimab | 28 | 0.652 (0.342 ~ 1.242) | 0.193 | 0.852 (0.315 ~ 2.304) | 0.752 |
| toripalimab | 3 | 1.317 (0.311 ~ 5.570) | 0.708 | 0.000 (0.000 ~ Inf) | 0.998 |

**Supplementary Table S4.** Adverse events in combination group after matching.

| Adverse events | All grades | Grade-1/2 | Grade-3/4 | Grade 5 |
|---|---|---|---|---|
| *TACE-related* | | | | |
| Fatigue | 2(1.6%) | 2(1.6%) | 0 | 0 |
| Hepatic impairment | 56(44.1%) | 54(42.5%) | 2(1.6%) | 0 |
| Abdominal pain | 26(20.5%) | 26(20.5%) | 0 | 0 |
| Gastrointestinal reaction | 29(22.8%) | 27(21.3%) | 2(1.6%) | 0 |
| Pyrexia | 18(14.2%) | 14(11.0%) | 2(1.6%) | 0 |
| Decreased WBC count | 1(0.8%) | 1(0.8%) | 0 | 0 |
| *ICIs-related* | | | | |
| Fatigue | 8(6.3%) | 8(6.3%) | 0 | 0 |
| Hepatic impairment | 2(1.6%) | 2(1.6%) | 0 | 0 |
| Gastrointestinal reaction | 8(6.3%) | 8(6.3%) | 0 | 0 |
| Pyrexia | 4(3.1%) | 4(3.1%) | 0 | 0 |
| Thrombocytopenia | 5(3.9%) | 4(3.1%) | 1(0.8%) | 0 |
| Decreased WBC count | 2(1.6%) | 2(1.6%) | 0 | 0 |
| Skin toxicity | 12(9.4%) | 10(7.9%) | 2(1.6%) | 0 |
| Proteinuria | 2(1.6%) | 2(1.6%) | 0 | 0 |
| Hypertension | 4(3.1%) | 4(3.1%) | 0 | 0 |
| Hypothyroidism | 3(2.4%) | 2(1.6%) | 1(0.8%) | 0 |
| *Donafenib-related* | | | | |
| Fatigue | 2(1.6%) | 2(1.6%) | 0 | 0 |
| Hepatic impairment | 4(3.1%) | 2(1.6%) | 2(1.6%) | 0 |
| Gastrointestinal reaction | 13(10.2%) | 12(9.4%) | 1(0.8%) | 0 |
| Thrombocytopenia | 1(0.8%) | 1(0.8%) | 0 | 0 |
| Decreased WBC count | 2(1.6%) | 2(1.6%) | 0 | 0 |
| Skin toxicity | 50(39.4%) | 45(35.4%) | 5(3.9%) | 0 |
| Proteinuria | 1(0.8%) | 1(0.8%) | 0 | 0 |
| Hypertension | 15(11.8%) | 15(11.8%) | 0 | 0 |

Gastrointestinal reaction includes nausea, vomiting, and diarrhea.
Hepatic impairment includes increased AST, increased ALT, and elevated bilirubin.

**Supplementary Table S5.** Adverse events in monotherapy group after matching.

| Adverse events | All grades | Grade-1/2 | Grade-3/4 | Grade 5 |
|---|---|---|---|---|
| Fatigue | 2(1.6%) | 2(1.6%) | 0 | 0 |
| Hepatic impairment | 86(67.7%) | 81(63.8%) | 5(3.9%) | 0 |
| Abdominal pain | 44(34.6%) | 43(33.9%) | 1(0.8%) | 0 |
| Gastrointestinal reaction | 19(15.0%) | 19(15.0%) | 0 | 0 |
| Pyrexia | 25(19.7%) | 23(18.1%) | 2(1.6%) | 0 |
| Thrombocytopenia | 13(10.2%) | 12(9.4%) | 1(0.8%) | 0 |

Gastrointestinal reaction includes nausea, vomiting, and diarrhea.

Hepatic impairment includes increased AST, increased ALT, and elevated bilirubin.

**Supplementary Table S6.** The baseline characteristics of the unmatched patients.

| Characteristics | unmatched patients (n = 110) | matched patients (n = 254) | *p* value |
|---|---|---|---|
| Age, Mean ± SD | 59.86 ± 8.73 | 60.04 ± 11.10 | 0.874 |
| Gender, n (%) | | | |
| male | 95 (86.36) | 207 (81.50) | 0.257 |
| female | 15 (13.64) | 47 (18.50) | |
| Child-pugh, n (%) | | | 0.311 |
| A | 96 (87.27) | 211 (83.07) | |
| B | 14 (12.73) | 43 (16.93) | |
| Cirrhosis, n (%) | | | 0.356 |
| no | 36 (32.73) | 96 (37.80) | |
| yes | 74 (67.27) | 158 (62.20) | |
| HBV, n (%) | | | 0.297 |
| no | 27 (24.55) | 50 (19.69) | |
| yes | 83 (75.45) | 204 (80.31) | |
| Tacetype, n (%) | | | 0.001 |
| cTACE | 76 (69.09) | 213 (83.86) | |
| DEB-TACE | 34 (30.91) | 41 (16.14) | |
| ALT, n (%) | | | 0.775 |
| ＜50U/L | 75 (68.18) | 177 (69.69) | |
| ≥50U/L | 35 (31.82) | 77 (30.31) | |
| AST, n (%) | | | 0.075 |
| ＜40U/L | 46 (41.82) | 132 (51.97) | |
| ≥40U/L | 64 (58.18) | 122 (48.03) | |
| TBIL, n (%) | | | 0.082 |
| ＜20μmol/L | 65 (59.09) | 174 (68.50) | |
| ≥20μmol/L | 45 (40.91) | 80 (31.50) | |
| ALB, n (%) | | | 0.001 |
| ＜40g/L | 72 (65.45) | 119 (46.85) | |
| ≥40g/L | 38 (34.55) | 135 (53.15) | |
| AFP, n (%) | | | 0.067 |
| ＜400ng/ml | 90 (81.82) | 185 (72.83) | |
| ≥400ng/ml | 20 (18.18) | 69 (27.17) | |
| Up-to-seven criteria, n (%) | | | 0.296 |
| within | 27 (24.55) | 76 (29.92) | |
| beyond | 83 (75.45) | 178 (70.08) | |

**Supplementary Fig. S1.** Absolute standardized mean difference of propensity score matching.

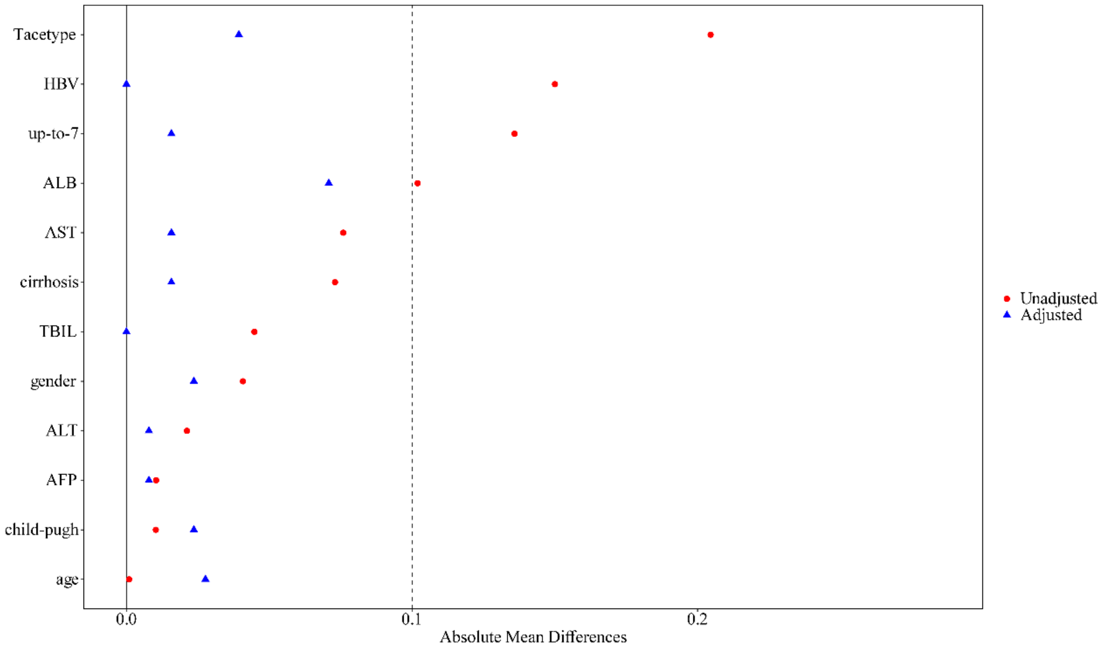


Note: post-matching standardised mean differences (SMD) < 0.1.

**Supplementary Fig. S2.** Density Plots before and after propensity score matching.

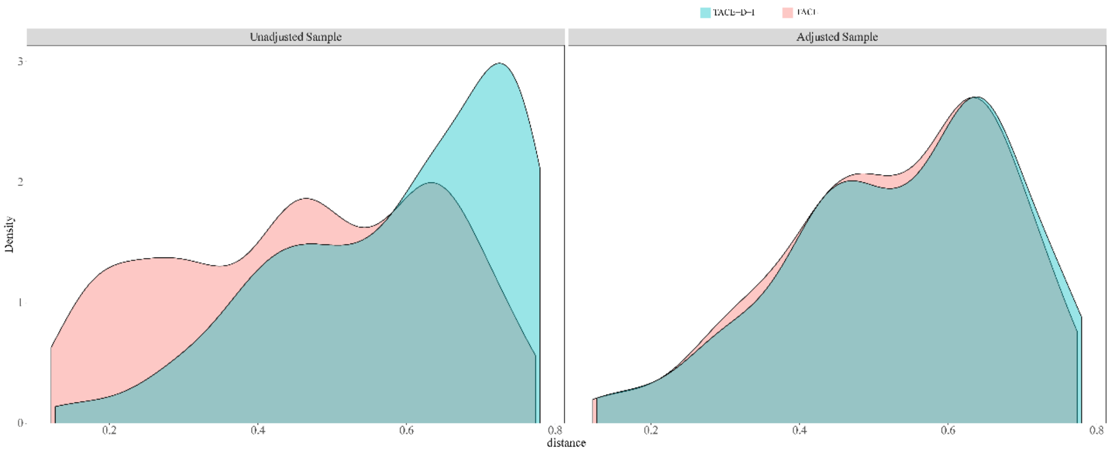

**Supplementary Fig. S3.** Kaplan–Meier analysis of progression-free survival (a) and overall survival (b) before matching.

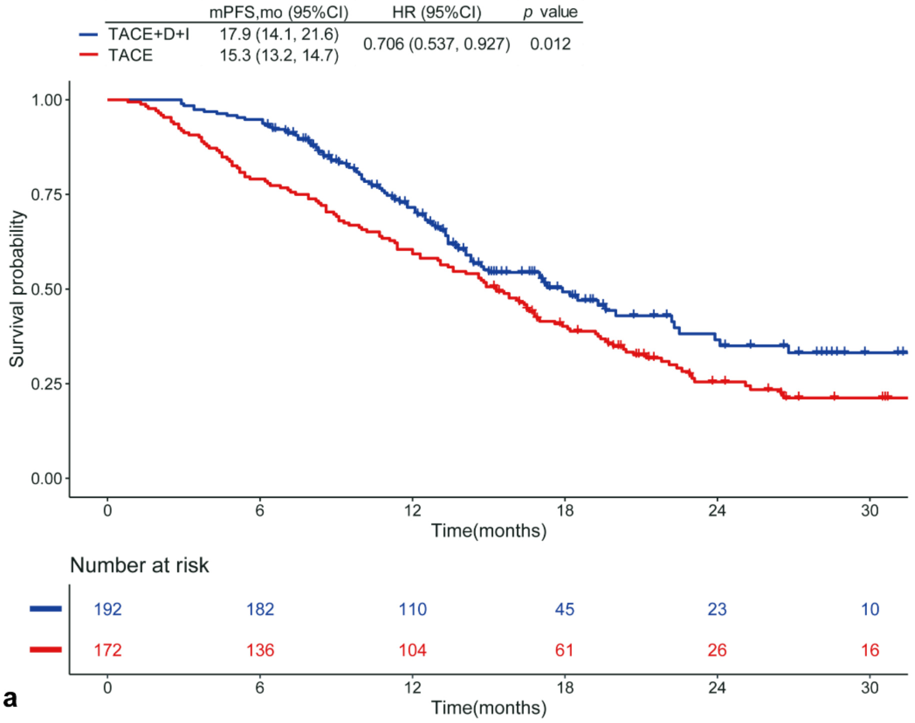


a

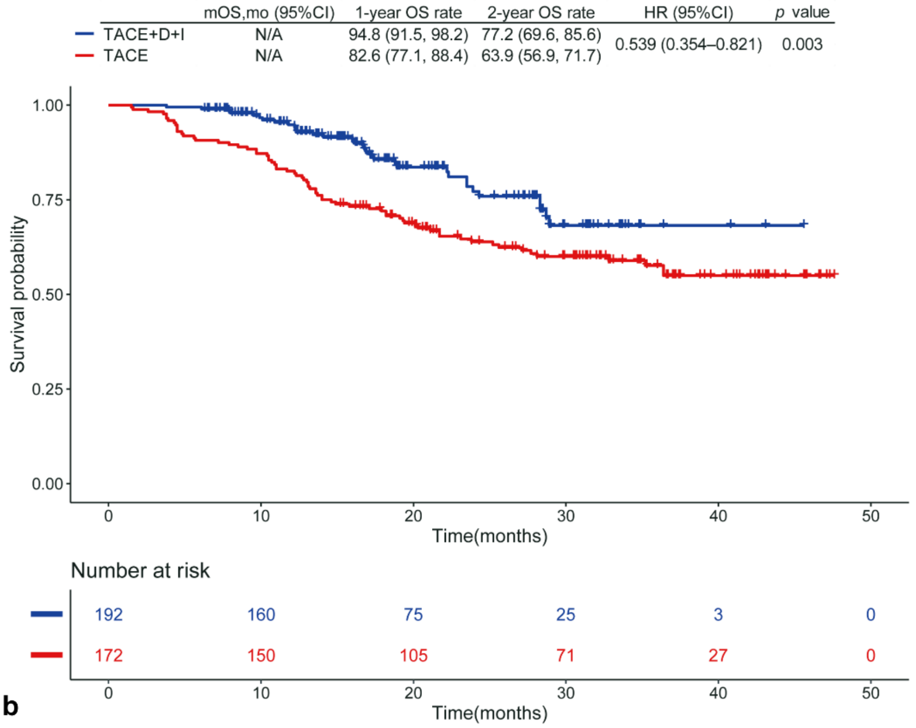


b

,

**Supplementary Fig. S4.** Kaplan–Meier analysis of progression-free survival for subgroup analysis based on baseline tumor burden after matching.

**a** for the subgroup beyond the up-to-seven criteria. **b** for the subgroup within the up-to-seven criteria.

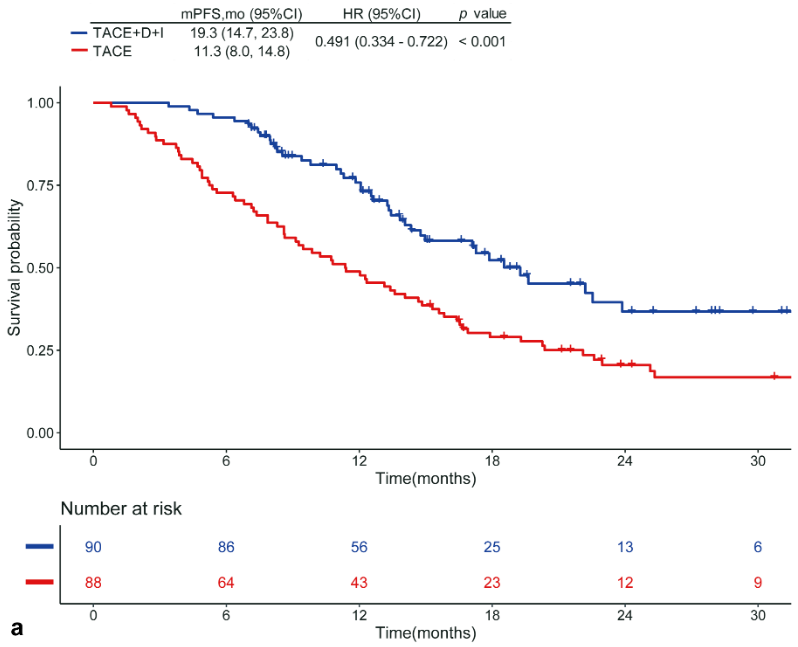


a

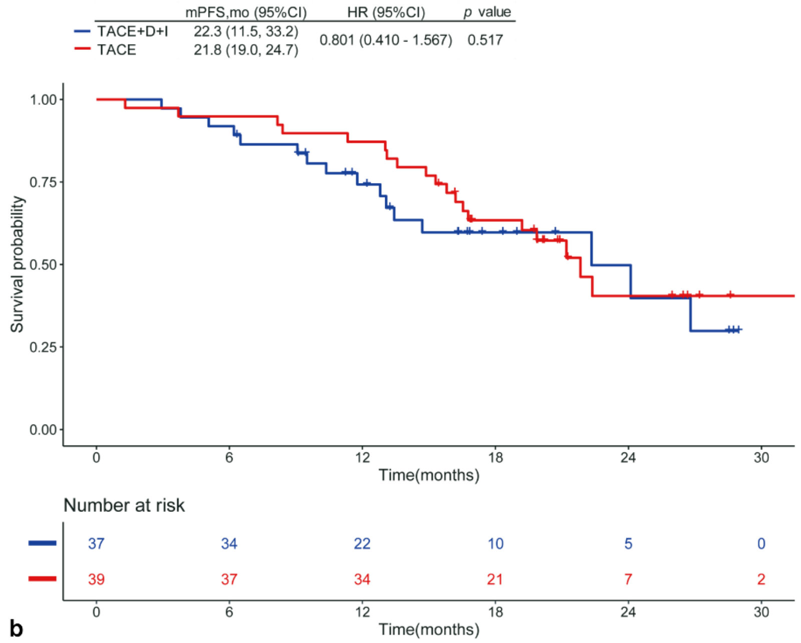


b

**Supplementary Fig. S5.** Kaplan–Meier analysis of progression-free survival for sensitivity analysis.

**a** for matching at a ratio of 2:1. **b** for matching with several key clinical factors.

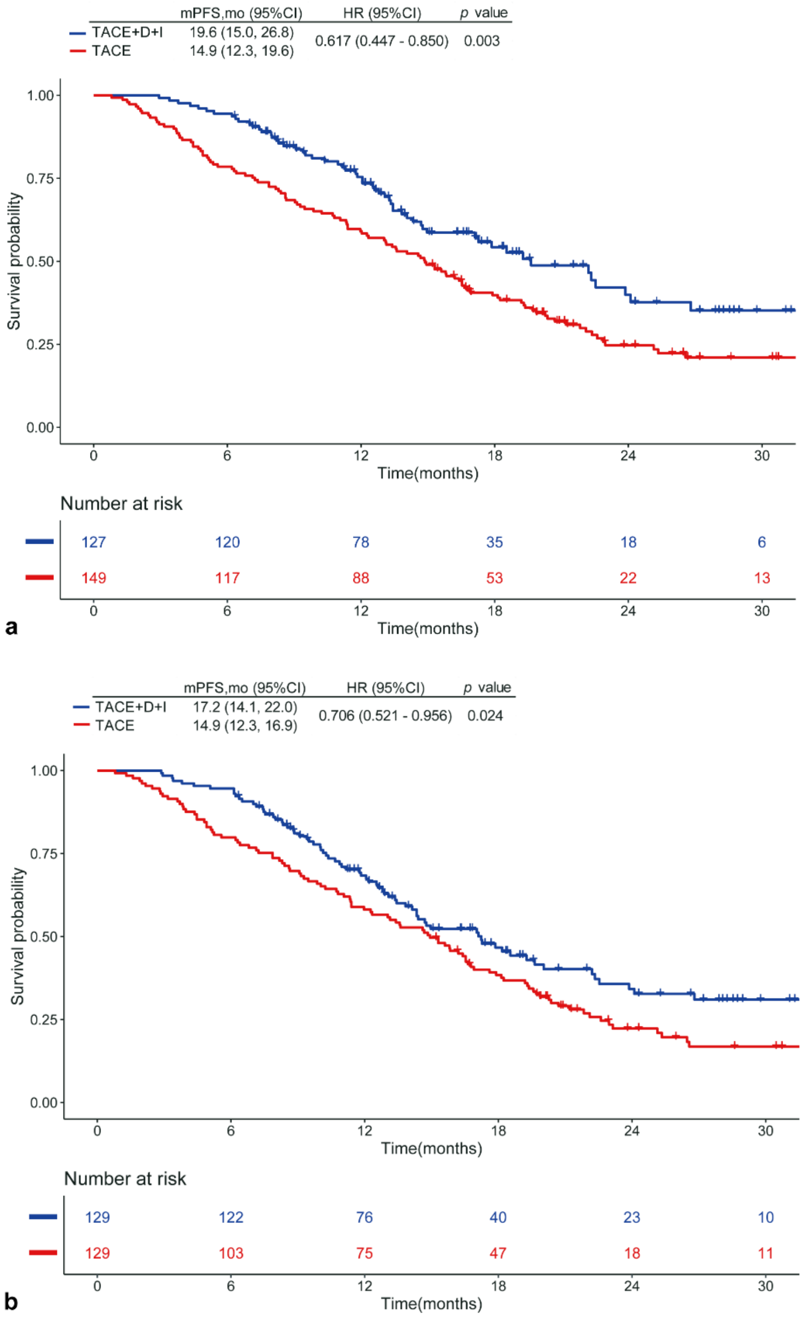

## Reference


1 Zhu H-D, Liu R, Jia Z-Z et al (2024) Transarterial chemoembolization for hepatocellular carcinoma: Treatment algorithm proposed by Chinese College of Interventionalists (CCI). EngMedicine 1:100037. https://doi.org/10.1016/j.engmed.2024.100037

2 Clinical Guidelines Committee of Chinese College of I (2023) [Chinese clinical practice guidelines for transarterial chemoembolization of hepatocellular carcinoma (2023 edition)]. Zhonghua Yi Xue Za Zhi 103:2674-2694. https://doi.org/10.3760/cma.j.cn112137-20230630-01114

3 Lu J, Zhao M, Arai Y et al (2021) Clinical practice of transarterial chemoembolization for hepatocellular carcinoma: consensus statement from an international expert panel of International Society of Multidisciplinary Interventional Oncology (ISMIO). Hepatobiliary Surg Nutr 10:661-671. https://doi.org/10.21037/hbsn-21-260

4 Llovet JM, Villanueva A, Marrero JA et al (2021) Trial Design and Endpoints in Hepatocellular Carcinoma: AASLD Consensus Conference. Hepatology 73 Suppl 1:158-191. https://doi.org/10.1002/hep.31327

5 Zhu HD, Li HL, Huang MS et al (2023) Transarterial chemoembolization with PD-(L)1 inhibitors plus molecular targeted therapies for hepatocellular carcinoma (CHANCE001). Signal Transduct Target Ther 8:58. https://doi.org/10.1038/s41392-022-01235-0

## THE CHANCE2410 INVESIGATORS

Rong Ding[1,2,3,4,5*], Xiao-Yang Xu[6,7*], Rui-Bao Liu[8*], Jun Tie[9*], Xu-Hua Duan[10], Bin Xiong[11], Deng-Gao Yuan[12], Wei-Jun Fan[13], Lu Wang[14], Zhi-Qiang Wu[15], Jie Zheng[16], Hui Zhao[17], Chang-Long Hou[18], Jin-Long Song[19], Ben-Sheng Zhao[20], Xiao-Li Zhu[7], Yong-Jie Su[21], Song Wang[22], Guo-Wen Yin[23], You Lu[23], Qing-Yu Xu[23], Hao Jiang[23], Qing-Qiao Zhang[24], Wei-Dong Wang[25], Peng Song[26], Bo-Gen Ye[27], Zhong-Wei Zhao[28], Feng-Zheng Zhang[29], Zhi-Qiang Fu[30], Chan Xie[31], Hai-Min Chen[32], Qing-He Tang[33], Ping Guo[34], Ming-Xin Pan[35], Jin-Hua Song[36], Wei-Zhong Zhou[37], Chun Lv[38], Shan-Zhi Gu[39], Kang-Shun Zhu[40], Ming-Rong Cao[41], Ning Huang[42], Hong-Wen Zhang[43], Qing-Han Li[44], Li Chen[1,2,3,4], Hai-Dong Zhu[1,2,3,4#], Gao-Jun Teng[1,2,3,4#], Bin-Yan Zhong[45#]

1. Center of Interventional Radiology and Vascular Surgery, Nurturing Center of Jiangsu Province for State Laboratory of AI Imaging & Interventional Radiology (Southeast University), Department of Radiology, Zhongda Hospital, Medical School, Southeast University, 87 Dingjiaqiao Road, Nanjing 210009, China.
2. National Innovation Platform for Integration of Medical Engineering Education (NMEE) (Southeast University), Nanjing 210009, China.
3. Basic Medicine Research and Innovation Center of Ministry of Education, Zhongda Hospital, Southeast University, Nanjing 210009, China.
4. State Key Laboratory of Digital Medical Engineering, Southeast University, Nanjing 210009, China.
5. Department of Minimally Invasive Intervention, Yunnan Cancer Hospital, The Third Affiliated Hospital of Kunming Medical University, Kunming 650106, China.
6. Department of Vascular Surgery and Interventional Department, The Fourth Affiliated Hospital of Soochow University, Dushu Lake Hospital, Suzhou 215006, China.
7. Department of Interventional Radiology, The First Affiliated Hospital of Soochow University, Suzhou 215006, China.
8. Department of Interventional Radiology, The Tumor Hospital of Harbin Medical University, Harbin 150081, China.
9. National Clinical Research Center for Digestive Diseases and Xijing Hospital of Digestive Diseases, Air Force Medical University, Xi'an 710000, China.
10. Department of Interventional Radiology, The First Affiliated Hospital of Zhengzhou University, Zhengzhou 450052, China.
11. Department of Interventional Radiology, The First Affiliated Hospital, College of Medicine, Zhejiang University, Hangzhou 310003, China.
12. Ningbo No.2 Hospital, University of Chinese Academy of Sciences Ningbo Huamei Hospital, Ningbo 315099, China.
13. Department of Imaging and Intervention, Cancer Center, Sun Yat-sen University, State Key Laboratory of Oncology in Southern China, Guangzhou 510060, China.
14. Fudan University Shanghai Cancer Center, Shanghai 200032, China.
15. Department of Interventional Radiology, The First Affiliated Hospital of Sun Yat-sen University, Guangzhou 510080, China.
16. Department of Interventional Radiology, The First Affiliated Hospital of Wenzhou Medical University, Wenzhou 325000, China.
17. Department of Interventional & Vascular Surgery, Affiliated Hospital of Nantong University, Medical

School of Nantong University, Nantong 226001, China.
18. Department of Interventional Radiology, Anhui Provincial Hospital, the First Affiliated Hospital of the University of Science and Technology of China, Hefei 230001, China.
19. Department of Surgical Oncology (Interventional Therapy), Shandong Tumor Hospital and Institute, Jinan, 250117, China.
20. Department of Interventional Radiology, The First Affiliated Hospital of Anhui Medical University, Hefei, Anhui 230022, China.
21. Department of Hepatobiliary Surgery, Zhongshan Hospital of Xiamen University, Fujian Provincial Key Laboratory of Chronic Liver Disease and Hepatocellular Carcinoma, Xiamen 361004, China.
22. Department of Interventional Radiology, The Affiliated Hospital of Qingdao University, Qingdao 266000, China.
23. Department of Interventional Radiology, Jiangsu Cancer Hospital & Jiangsu Institute of Cancer Research & The Affiliated Cancer Hospital of Nanjing Medical University, Nanjing, 210009, China.
24. Department of Interventional Radiology, the Affiliated Hospital of Xuzhou Medical University, Xuzhou 221006, China.
25. Department of Interventional Radiology, The Affiliated Wuxi People's Hospital of Nanjing Medical University, Wuxi 214023, China.
26. Department of Interventional Therapy, National Cancer Center/National Clinical Research Center for Cancer/Cancer Hospital and Shenzhen Hospital, Chinese Academy of Medical Sciences and Peking Union Medical College, Shenzhen 518100, China.
27. Renji Hospital, Shanghai JiaoTong University School of Medicine, Shanghai 200127, China.
28. Department of Interventional Radiology, Zhejiang University Lishui Hospital, The Fifth Affiliated Hospital of Wenzhou Medical University, Lishui Central Hospital, Lishui 323000, China.
29. Department of Interventional Radiology, Anhui Provincial Hospital, The First Affiliated Hospital of University of Science and Technology of China (USTC), Hefei 230001, China.
30. Department of Hepatobiliary Surgery, Sun Yat-sen Memorial Hospital, Sun Yat-sen University, Guangzhou 510120, China.
31. The Third Affiliated Hospital of Sun Yat-sen University, Guangzhou 510120, China.
32. Yuebei People's Hospital, Shaoguan 512026, China.
33. Department of Biliary and Pancreatic Surgery, East Hospital Affiliated to Tongji University in Shanghai, Shanghai 200120, China.
34. The First Affiliated Hospital of Xiamen University, School of Medicine, Xiamen University, Xiamen 361102, China.
35. Zhujiang Hospital of Southern Medical University, Guangzhou 510280, China.
36. Hepatobiliary Center, Jiangsu Provincial People's Hospital, Nanjing 210029, China.
37. Department of Interventional Radiology, Jiangsu Provincial People's Hospital, Nanjing 210029, China.
38. Huai'an No.4 People's Hospital, Huai’an 223002, China.
39. Department of Interventional Therapy, Hunan Cancer Hospital and the Affiliated Cancer Hospital of Xiangya School of Medicine, Central South University, Changsha 410013, China.
40. Department of Interventional Radiology, The Second Affiliated Hospital of Guangzhou Medical University, Guangzhou 510145, China.
41. The First Affiliated Hospital of Jinan University, Guangzhou Overseas Chinese Hospital, Guangzhou 510630, China.
42. Fujian Medical University Union Hospital, Fuzhou 350001, China.

43. The 900 Hospital of the Joint Service Support Force of the People's Liberation Army of China, Fuzhou 350025, China.
44. Department of Hepatobiliary Surgery, First People's Hospital of Foshan, Foshan 528000, China.
45. Department of Interventional Radiology, Zhejiang Cancer Hospital, Hangzhou Institute of Medicine (HIM), Chinese Academy of Sciences, Hangzhou 310022, China.
